# Protein degradation by a component of the chaperonin‐linked protease ClpP

**DOI:** 10.1111/gtc.13141

**Published:** 2024-07-04

**Authors:** Fumihiro Ishikawa, Michio Homma, Genzoh Tanabe, Takayuki Uchihashi

**Affiliations:** ^1^ Faculty of Pharmacy Kindai University Osaka Japan; ^2^ Department of Biomolecular Engineering, Graduate School of Engineering Nagoya University Nagoya Japan; ^3^ Division of Material Science, Graduate School of Science Nagoya University Nagoya Japan

**Keywords:** ATPase, ClpX, *E. coli*, Lon, proteasome

## Abstract

In cells, proteins are synthesized, function, and degraded (dead). Protein synthesis (spring) is important for the life of proteins. However, how proteins die is equally important for organisms. Proteases are secreted from cells and used as nutrients to break down external proteins. Proteases degrade unwanted and harmful cellular proteins. In eukaryotes, a large enzyme complex called the proteasome is primarily responsible for cellular protein degradation. Prokaryotes, such as bacteria, have similar protein degradation systems. In this review, we describe the structure and function of the ClpXP complex in the degradation system, which is an ATP‐dependent protease in bacterial cells, with a particular focus on ClpP.

## Graphical abstract

Systems that degrade polymeric amino acids are essential for life. In this study, we introduce the structural and functional analyses of the ClpP protease, which is mainly responsible for selective proteolysis in microorganisms, and explain its roles and control mechanisms.

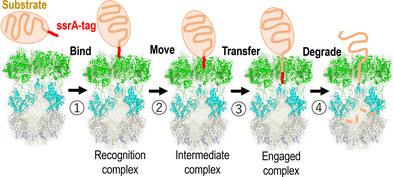

## 1 INTRODUCTION

Protein homeostasis (proteostasis) is maintained by the balance between protein synthesis and degradation. Protein synthesis is primarily controlled by transcription and translation, whereas protein degradation is mediated by various proteases. The ubiquitin‐proteasome and autophagy‐lysosome systems exist in the cytoplasm of eukaryotes and are responsible for protein degradation (Varshavsky, 2017). The former is a large protein complex that recognizes and degrades ubiquitinated substrate proteins (Bard et al., 2018). In the latter system, substrate proteins present in the cytoplasm are taken up by membrane vesicles and fused with lysosomes that contain proteases that degrade the proteins (Mizushima et al., 2011). In contrast, prokaryotes have energy‐dependent proteolytic systems such as a complex consisting of Lon, FtsH, ClpXP, ClpAP, and HslUV (Gottesman, 1996; Gur et al., 2011; Mahmoud & Chien, 2018; Olivares et al., 2016). The *lon* gene was identified in a mutant that gave the phenotype sensitive to UV light in *Escherichia coli*. The *lon* gene product is an ATP‐dependent proteolytic enzyme La (Chung & Goldberg, 1981; Charette et al., 1981). The Lon (La) protease is involved in the degradation of proteins with abnormal conformations resulting from nonsense or missense mutations, synthetic errors, or intracellular denaturation (Goldberg, 1992). The Lon protease degrades SulA (a protein that inhibits cell division) and is involved in the SOS response (Mizusawa & Gottesman, 1983). The *ftsH* gene has been identified as a temperature‐sensitive and cell‐division‐defective mutant and is a membrane protein with two transmembrane regions belonging to the AAA+ family (Ito & Akiyama, 2005). FtsH is a metalloprotease with a Zn^2+^ binding site that is primarily responsible for the degradation of membrane proteins. A protein degradation system similar to the proteasome in eukaryotes is the Clp protease system in prokaryotes, such as Archaea or Bacteria containing Actinobacteria or Mycobacteria (Becker & Darwin, 2017; Humbard & Maupin‐Furlow, 2013). In *E. coli*, ClpXP has been biochemically identified as an ATP‐dependent protease, Ti (Clp), which differs from Lon (Hwang et al., 1987; Katayama‐Fujimura et al., 1987). These prokaryotic Clp systems are involved in protein degradation, contain ATPases belonging to the AAA+ family, and share several structural similarities. Subsequently, a heat shock protein called HslVU was identified as an ATP‐dependent protease (Gottesman et al., 1993) and was also called ClpYQ because it was shown to be similar to the Clp protease. The ClpYQ complex has a 4‐ring structure similar to that of the eukaryotic 26S proteasome and a 6‐fold symmetric ring structure with strong homology to the 20 proteasome subunits (Kessel et al., 1996; Rohrwild et al., 1997, p. 223). All protease systems are important for sustaining life; however, in this review, we discuss their functions, structures, and roles in bacteria, focusing on ClpP, which has protease activity, and its regulation of cell function by the selective degradation of certain proteins, in the research of which the authors are directly involved.

## 2 FUNCTION OF CLP PROTEASE

**FIGURE 1:**
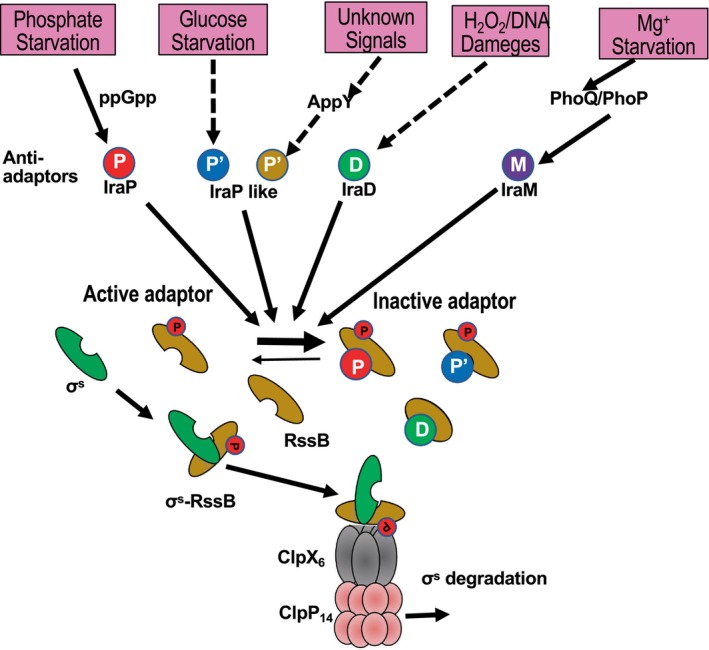
Model of σ^s^ turnover control by anti‐adapter proteins involving ClpXP (modified from reference; Bougdour et al., 2008). In logarithmically growing bacteria (*Escherichia coli*), σ^s^ (green oval) is loaded onto ClpX (gray) by RssB (ocher oval) and rapidly degraded by ClpP (pink). Anti‐adapter proteins (IraP, IraM, IraD; green, red, purple) inhibit the σs degradation through direct interactions with RssB. When the anti‐adapter protein binds to RssB, RssB becomes not to be able to bind σ^s^, and σ^s^ is no longer sent to ClpXP. Each anti‐adapter protein is produced under specific stresses or starvation conditions. When *E. coli* cells are placed under a phosphate‐starved condition, ppGpp accumulates and the transcription of *iraP* is activated. Under the Mg^2+^ starvation condition, the PhoP/PhoQ regulatory system senses the starvation signals, and this information is transmitted to the *iraM* gene in *E. coli*. Synthesis of IraM inhibits the proteolysis of σ^s^. In *E. coli*, the *iraD* expression is induced in the cells that were subjected to hydrogen peroxide or DNA damage stress, and the degradation of σ^s^ is suppressed (Zheng et al., 2001). Phosphorylation of RssB (red circle) enhances its interaction with σ^s^ but is not an absolute requirement for its control. Even in the absence of phosphorylation of RssB, RssB assumes to take the “on” conformation.

The Clp proteolytic system is an AAA+ chaperone‐protease complex that contains **A**TPases **a**ssociated with diverse cellular **a**ctivities that assist the structural changes present in both eukaryotes and prokaryotes. However, in eukaryotes, it is present in mitochondria or chloroplasts, which are evolutionarily derived from prokaryotes. The Clp system is involved in the transcription and regulation of bacterial growth. A typical example of ClpXP is shown in Figure 1 referred from a previous report (Bougdour et al., 2008). More general roles of ClpXP are introduced in a recent review (Bouillet et al., 2024). The Clp protease complex comprises a hexameric ring, which is an unfolding ATPase with a chaperone function that introduces protein substrates into the protease complex, and a tetradecamer ring in which the heptameric rings overlap and have serine protease activity with adapter proteins. ClpP is a component of the tetradecamer ring that exhibits proteolytic enzymatic activity and is highly conserved among microbial species. However, homology among unfoldases, such as ClpX, which has chaperone functions, and ATPases, is low. Bacteria have different unfoldase ATPases, namely, ClpX, ClpA, ClpC, and ClpE. A wide variety of systems are responsible for the decomposition of proteins, and some examples are shown below. Substrate proteins are involved in many intracellular pathways, such as the DNA damage response, metabolism, and transcriptional regulation, and their degradation regulates many intracellular functions.

In *E. coli*, ClpXP interacts with SspB or ClpAP, which interacts with ClpS, and SspB and ClpS function as adapter proteins to degrade proteins tagged with SsrA (Kirstein et al., 2009). Regarding the SsrA tag, *ssrA* is transcribed as a tmRNA and recognized as an mRNA by tRNA synthetase in the ribosome, and peptide addition occurs via translation (Withey & Friedman, 2003). When this peptide (SsrA tag) is recognized by ClpX and ClpA (unfoldase ATPases), the attached proteins are translocated to ClpP, where they are degraded into small peptides (Fritze et al., 2020).

Sigma factor RpoS (σ^s^), a subunit of RNA polymerase, increases the expression of stress‐responsive genes in *E. coli* (Bouillet et al., 2024). Although ClpXP was involved in the turnover of σ^s^, RssB (Regulator of Sigma S) was required as an adapter for the decomposition of σ^s^. RssB, a two‐component response regulator, has a CheY (a signal transduction protein of chemotaxis system in motility by flagella)‐like a phosphorylation site, and phosphorylation allows it to bind to σ^s^. ClpX recognizes the RssB‐σ^s^ complex and sends it to ClpP to degrade it (Hengge, 2009). An anti‐adapter protein (an inhibitor of RssB activity, Ira) has been identified as a binding factor that inhibits RssB degradation. Ira proteins are responsible for regulating signals such as phosphate starvation, magnesium starvation, and DNA damage, and the corresponding anti‐adapter proteins, IraP, IraM, and IraD, have been identified (Bougdour et al., 2008; Hengge, 2009; Kirstein et al., 2009) (Figure 1).

In *Bacillus subtilis*, ComK is the main regulator of competence gene transcription and is antagonized by the adaptor protein MecA. MecA not only inhibits ComK activity directly but also requires the chaperone function of ClpC as an adapter protein for the recognition of ComK, which is degraded by the ClpCP complex (Elsholz et al., 2017; Schlothauer et al., 2003). The arginine kinase McsB is also involved in degradation by *B. subtilis* ClpCP (Trentini et al., 2016). ClpC binds to the phosphorylated arginine residues and delivers them to ClpP.

In *Salmonella enterica*, FlhDC is degraded by ClpXP, because FlhDC molecules accumulate in ClpX‐deficient *Salmonella* strain (Tomoyasu et al., 2003). FlhD and FlhC form the FlhD_4_C_2_ complex, which acts as a master transcriptional regulator of flagellar formation genes in *E. coli* and *Salmonella*. In in vitro studies, the YdiV adapter protein is involved in the regulation of FlhDC degradation by ClpXP (Takaya et al., 2012). Although the crystal structures of the YdiV and FlhD complexes have been elucidated, it is unclear how they function as adapter proteins for degradation by ClpXP (Li et al., 2012). Furthermore, FliT, which negatively regulates the transcriptional activity of FlhDCs and acts as a chaperone for flagellar transport proteins, specifically promotes degradation by ClpXP (Sato et al., 2014). It was speculated that FliT assists in the recognition of FlhC by ClpX because no direct interaction between FliT and ClpX was detected.

## 3 GENES OF CLP PROTEASE

As mentioned above, proteases other than Lon (La) have been identified as Ti(Clp), which are also ATP‐dependent proteases (Hwang et al., 1987; Katayama‐Fujimura et al., 1987). Unlike the Lon protease, Clp comprises two distinct subunits of approximately 20 and 80 kDa, named ClpP and ClpA, respectively (Katayama et al., 1988). The *clpA* gene was first mapped to 19 min on the *E. coli* chromosome and was obtained from a phage clone. The *clpP* gene is not in the vicinity of *clpA* and was mapped at 10 min onto the *E. coli* chromosome, which was also cloned from a phage library (Maurizi et al., 1990). ClpP reacts with antibodies against yeast‐derived proteasomes (Tanaka et al., 1989), and its amino acid sequence shows high homology among proteases. The roles of ATP‐dependent proteases have been elucidated in a previous review (Gottesman & Maurizi, 1992). In certain situations or at a suitable time, these proteases selectively degrade functional or regulatory proteins in addition to degrading abnormal proteins.

**FIGURE 2:**
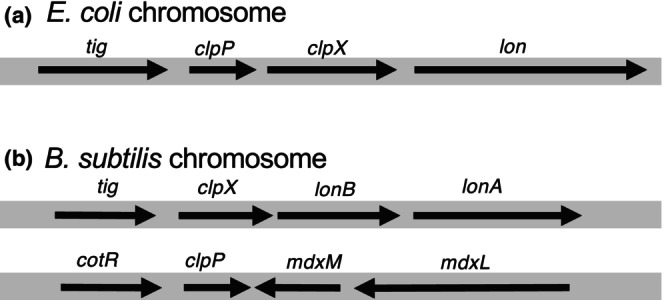
Gene organization of *clpX* and *clpP*. Genes encoding ClpX and ClpP proteins in *Escherichia coli* (a) or *Bacillus subtilis* (b). *tiRg*: Prolyl isomerase (trigger factor) gene; *clpP*: ATP‐dependent Clp protease proteolytic subunit; *clpX*: ATP‐dependent Clp protease ATP‐binding subunit; *lon*: ATP‐dependent protease Lon; *lonB*: Spore‐specific ATP dependent protease; *lonA*: class III heat shock ATP‐dependent protease.

The *E. coli* Clp protease consists of a hexameric ring composed of ClpX (or ClpA), an ATPase unfoldase with a chaperone function consisting of 424 amino acids, and a double heptameric ring (tetradecamer) composed of 207 amino acids with protease activity. As mentioned earlier, the *clpP* and *clpA* genes of *E. coli* are located separately on the chromosome, whereas the *clpX* gene is located downstream of the *clpP* gene and forms an operon (Figure 2a). Immediately downstream of the *clpP* gene is the ATP‐dependent protease *lon* is responsible for the degradation of misfolded proteins and many rapidly degraded regulatory proteins. In *B. subtilis*, *clpX* and *clpP* are located far apart and transcribed separately. These proteins are induced by heat shock in either *E. coli* or *B. subtilis*, the induction mechanisms are different and their transcription is controlled by different sigma factors in different gene organizations (Figure 2b). Some pathogenic bacteria, such as *Mycobacterium tuberculosis*, encode two *clpP* genes (*clpP1* and *clpP2*); ClpP1 and ClpP2 form a heptamer that combines to form a tetradecamer (Kahne & Darwin, 2021).

**FIGURE 3:**
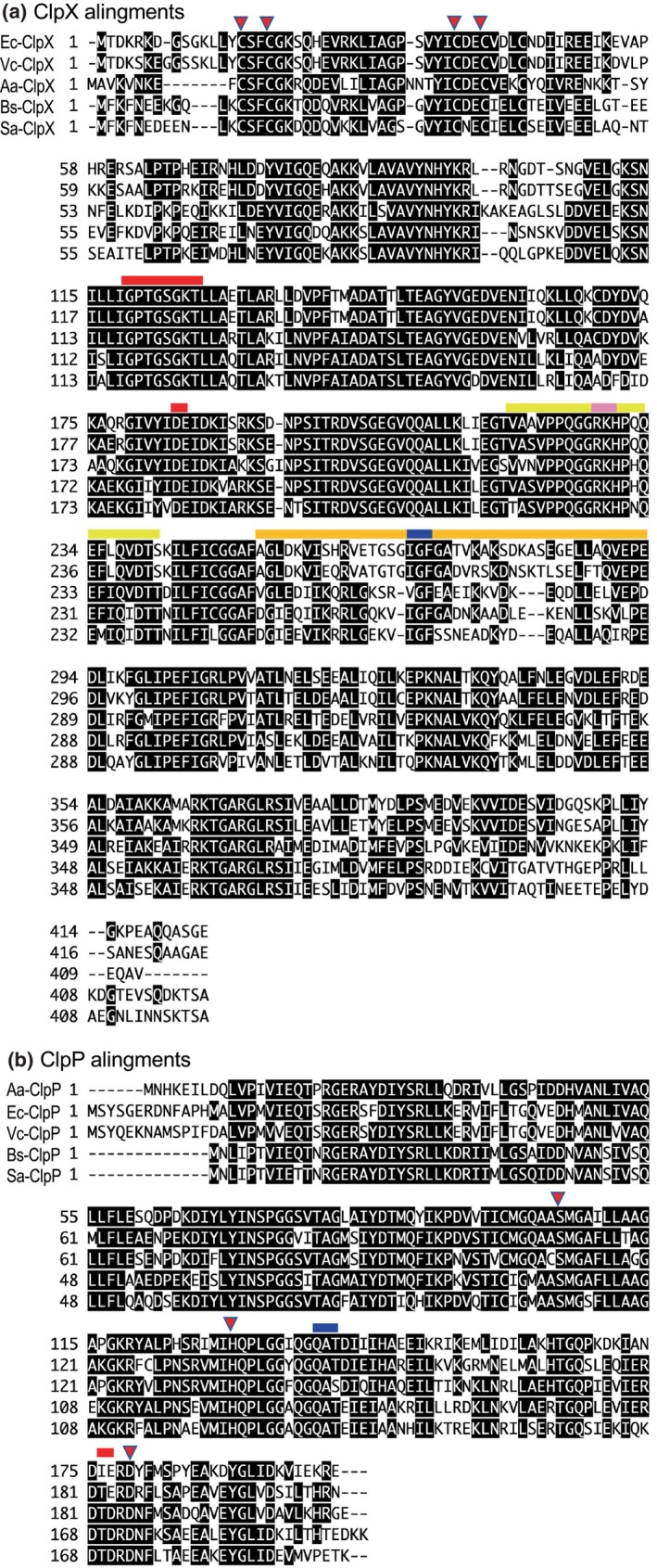
Amino acid sequence alignment of ClpX (a) and ClpP (b) from various bacteria. Amino acid residues with ≥50% homology in the five sequences are indicated by black boxes. Aa, *Aquifex aeolicus*; Ec, *Escherichia coli*; Vc, *Vibrio cholerae*, Bs, *Bacillus subtilis*; Sa, *Staphylococcus aureus*. (a) Cysteine residues involved in zinc binding (Cys‐14, Cys‐17, Cys‐36, Cys‐39) of *E. coli* are shown as a red triangle, the Waker A and Waker B motifs are shown as red bars, the IGF motif is shown as blue bars, the IGF loop region is shown as orange bars, the RKH motif is shown as pink bars, and the RKH loop region is shown as yellow. (b) QXT motif is shown as a blue bar, the catalytic triplet amino acid residues are shown as a red triangle, and the oligomerization sensor residue is shown as a red bar.

A comparison of the amino acid sequences of ClpP and ClpX revealed that they were homologous among bacteria, even between Gram‐negative and Gram‐positive bacteria (Figure 3). We speculate that the sequence of proteins had been almost completely adjusted for proteolytic function for survival, and the change or evolution of the protein seems unnecessary. On the other hand, by acquiring different ClpPs from different bacterial species, bacteria may adapt to the environment for survival.

**FIGURE 4:**
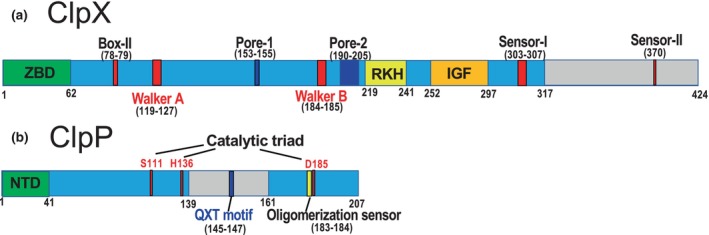
Model diagram of the *Escherichia coli* ClpP and ClpX amino acid sequences. (a) ClpX is composed of three major domains. (i) The N‐terminal zinc‐binding domain (ZBD), which is important for substrate recognition; (ii) the large AAA+ domain (light blue); and (iii) the small AAA+ domain (gray), which is responsible for ATP hydrolysis and forms a motor module. The large AAA+ domain contains Box‐II, Walker A, Walker B, and Sensor‐I Arginine finger (red), which are involved in ATP binding, and the pore‐1 loop, the pore‐2 loop, and RKH: Arg‐Lys‐His loop (blue) which are responsible for substrate recognition, unfolding, and translocation. Furthermore, this domain contains an IGF loop (orange) for binding to ClpP. The small AAA+ domain has a Sensor‐II arginine residue (pink) for ATP binding. (b) ClpP also has three major domains: (i) an N‐terminal motif important for ClpX interaction and axial pore regulation (green), (ii) a Ser‐His‐Asp catalytic triplet of amino acid residues (red) and the core domain (light blue) containing oligomerization sensor residues (yellow), and (iii) the handle domain (gray region sandwiched between the core domain sequences), which is important for the oligomerization of the two heptameric rings. The QXT(Glu‐X‐Thr) motif (blue), essential for stabilizing the interface between the two ClpP heptamers, is also located in the handle domain. Numbers indicate residue positions. This figure was based on the paper (Mabanglo & Houry, 2022).

ClpX is an ATPase consisting of approximately 400 amino acids that can be divided into three domains based on its sequence (Baker & Sauer, 2012) (Figure 4a). At the N‐terminus, a C4‐type zinc‐binding domain (ZBD) is important for substrate recognition, and the residues Cys‐14, Cys‐17, Cys‐36, and Cys‐39 in *E. coli* are involved in this binding (Wojtyra et al., 2003). The N‐terminal region is important for the dimerization of ClpX molecules. Next to this N‐terminal region, there are two AAA+ domains, large and small, which form the ATP hydrolysis and motor modules. The large and small AAA+ domains function together in hexameric rings, although the orientation of these domains can vary substantially between different subunits (Baker & Sauer, 2012). The large AAA+ domain contains Box‐II, Walker A, Walker B, and Sensor‐I Arginine fingers, which are involved in ATP‐dependent degradation; and pore‐1, pore‐2, and RKH, which are responsible for substrate recognition, unfolding, and translocation, respectively. The IGF (Ile‐Gly‐Phe) loop binds to ClpP. The small AAA+ domain contains a sensor II arginine residue for ATP binding. In contrast, ClpP contains three major domains: (i) an N‐terminal domain (NTD) with important motifs for ClpX interaction and axial pore regulation; (ii) a core domain containing the Ser‐His‐Asp catalytic triad and oligomerization sensor residues; and (iii) a handling domain important for the oligomerization of the two heptameric rings (Figure 4b) (Mabanglo & Houry, 2022). The QXT (Gln‐X‐Thr) motif, which is essential for stabilizing the interface between the two ClpP heptameric rings, is also located in the handle domain.

## 4 INHIBITORS OF CLP PROTEASE

**FIGURE 5:**
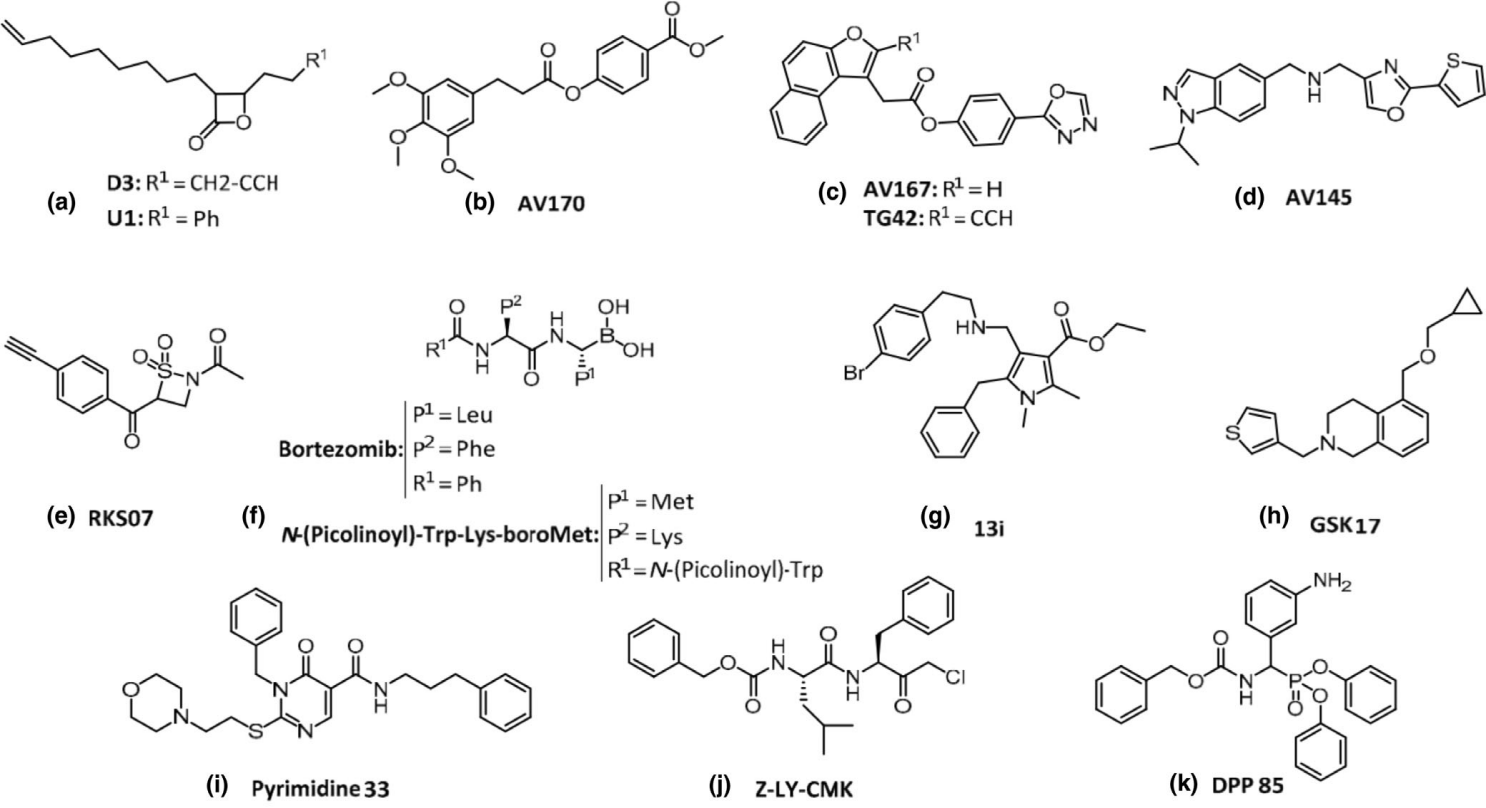
ClpP protease‐inhibitor compounds. (a) Lead compounds D3 and U1; (b) AV170; (c) AV167 and TG42; (d) AV145; (e) RKS07; (f) *N*‐(Picolinoyl)‐Trp‐Lys‐boroMet and Bortezomib; (g) 13i; (h) GSK17; (i) prymidine33; (j) Z‐LY‐CMK; (k) DPP85.

Inhibitors of Clp protease have been developed as antibacterial agents against pathogenic bacteria such as *Staphylococcus aureus* and *Listeria monocytogenes* (Moreno‐Cinos, Goossens, et al., 2019). The protease activity of ClpP is required for the expression of virulence genes in *S. aureus* (Frees et al., 2003). Because ClpP is a serine protease, classical approaches for designing drugs that can inhibit the catalytic site have been investigated; however, these have not been successful. Under such circumstances, in 2008 it was discovered that a compound containing β‐lactone acts on ClpP of *S. aureus* and weakens its toxicity (Böttcher & Sieber, 2008; Staub & Sieber, 2008). The lead compounds G2, E2, and D3 formed covalent bonds with the serine residue at the catalytic site of ClpP and irreversibly inhibited its activity. G2 showed the weakest effect on peptidase activity with an IC50 value of 31 μM, on the other hand, D3 and E2 showed a much stronger inhibitory effect and their IC50 values were 6 and 4 μM, respectively. D3 exhibited the strongest inhibitory effect on protease and hemolysin hemolysis (Figure 5a). From this compound, U1, which has higher antibacterial activity, was synthesized and by the peptidase activity assays using recombinant ClpP, EC50 of U1 showed 7 μM (Böttcher & Sieber, 2009). However, β‐lactone compounds are unstable electrophilic compounds and are quickly degraded in human plasma.

In the search for stable ClpP inhibitors, many compounds were screened, and six compounds were identified: a compound with five phenyl esters (AV126, AV168, AV127, AV167, and AV170) and a compound with a triazole amide (AV166) and the compounds showed the potent IC50 values between 0.3 and 1.3 μM (Hackl et al., 2015). Among these compounds, AV170 (Figure 5b) most strongly inhibits the protease activity of ClpP. Furthermore, AV167 (Figure 5c), which contains a naphthalene residue, strongly inhibits the protease activity of human‐derived ClpP (IC50 = ca. 1.5 μM). Furthermore, the compound TG42 (Figure 5c), in which the naphthalene moiety of AV167 was altered, lowered the IC_50_ against human ClpP by approximately 4 times (Gronauer et al., 2018). The first non‐covalent inhibitor of *S. aureus* ClpP, AV145 (Figure 5d) which IC50 was ca. 10 μM, was identified (Pahl et al., 2015). Binding of this compound fixes the structure via the conserved proline residue (P125), distorts the structure of the catalytic site, and inhibits the peptide bond cleavage activity of the protease. ClpP consists of two heptamers with no protease activity (Gersch et al., 2012). Based on the evidence, drugs with different protease inactivation mechanisms, diisopropyl fluorophosphate (DFP), β‐sultam RKS09, and β‐lactone E2, were developed to inhibit the formation of tetradecamer of ClpP (Gersch et al., 2014). In contrast, the β‐sultam compound (RKS07: Figure 5e), which IC50 is ca. 1.0 μM, does not change the oligomeric state of ClpP, and it has a strong protease inactivation effect to form a covalent bond with the serine residue in the catalytic site. This causes the irreversible inhibition of ClpP protease activity. Peptidomimetic boronate compounds inhibit the protease activity of *S. aureus* ClpP (Ju et al., 2020).

A high‐throughput screening system was constructed to measure protease activity in bacterial cells expressing *M. tuberculosis* casein‐degrading protease (ClpP1P2) and SssA‐tagged GFP protein in bacterial cells. Based on this screening system, the human 26S proteasome drug bortezomib was developed, which has been identified as a potent inhibitor of ClpP1P2 activity and bacterial growth (Moreira et al., 2015). Although it has been used clinically as an anticancer agent, it has not been used to treat the *M. tuberculosis* infection because of its high cost, weak pharmacokinetics, and short half‐life. The boronic acid derivative *N*‐(picolinoyl)‐Trp‐Lys‐boroMet (Figure 5f) exhibited high protease inhibitory activity (Akopian et al., 2015). Furthermore, several pyrrole core compounds such as ethyl 4‐(((4‐bromophenethyl) amino) methyl)‐2, 5‐dimethyl‐1‐phenyl‐1H‐pyrrole‐3‐carboxylate hydrochloride (13i: Figure 5g) were synthesized using docking simulations with compounds that inhibited the activity of ClpP1P2 and bacterial growth (Liu et al., 2018). In silico docking simulations were also performed, demonstrating the possibility of improving antibacterial activity. Furthermore, a new assay system that detects ATP‐dependent proteolysis of fluorescent proteins was used to conduct a large‐scale screening of compounds with antimicrobial activity against *M. tuberculosis*, and GSK17 (Figure 5h) and GSK18 were identified (Fraga et al., 2019). GSK18 is a new inhibitor of the ClpC protease through its ATPase inhibitory activity and showed IC50 of 42 ± 7 μM in the ATPase assay against ClpP1P2 peptidase activity; however, the mechanism of action of GSK17 is unknown. Screening for *Plasmodium* ClpP identified a novel ClpP inhibitor prymidine33 (Figure 5i), which has a pyrimidine ring and inhibits the growth and division of the apicoplast, leading to parasite death. Inhibitors of the gram‐negative bacterial ClpP remain largely undeveloped; benzyloxycarbonyl‐leucyltyrosine chloromethyl ketone (Z‐LY‐CMK: Figure 5j) (Powers et al., 1977), is the only known irreversible inhibitor of serine proteases such as subtilisin for ClpP in *E. coli*, and its co‐crystal structure has been solved (Szyk & Maurizi, 2006). Until recently, this drug was the only reported inhibitor of gram‐negative *E. coli* ClpP, but screening diaryl phosphonate‐based compounds led to the discovery of inhibitors; DPP85 (Figure 5k) which contains α‐aminodiarylphosphonic acid exhibits a low MIC concentration (IC_50_ = ca. 0.5 μM) and low cytotoxicity of *E. coli* (Moreno‐Cinos, Sassetti, et al., 2019).

## 5 ACTIVATORS OF ClpP PROTEASE

**FIGURE 6:**
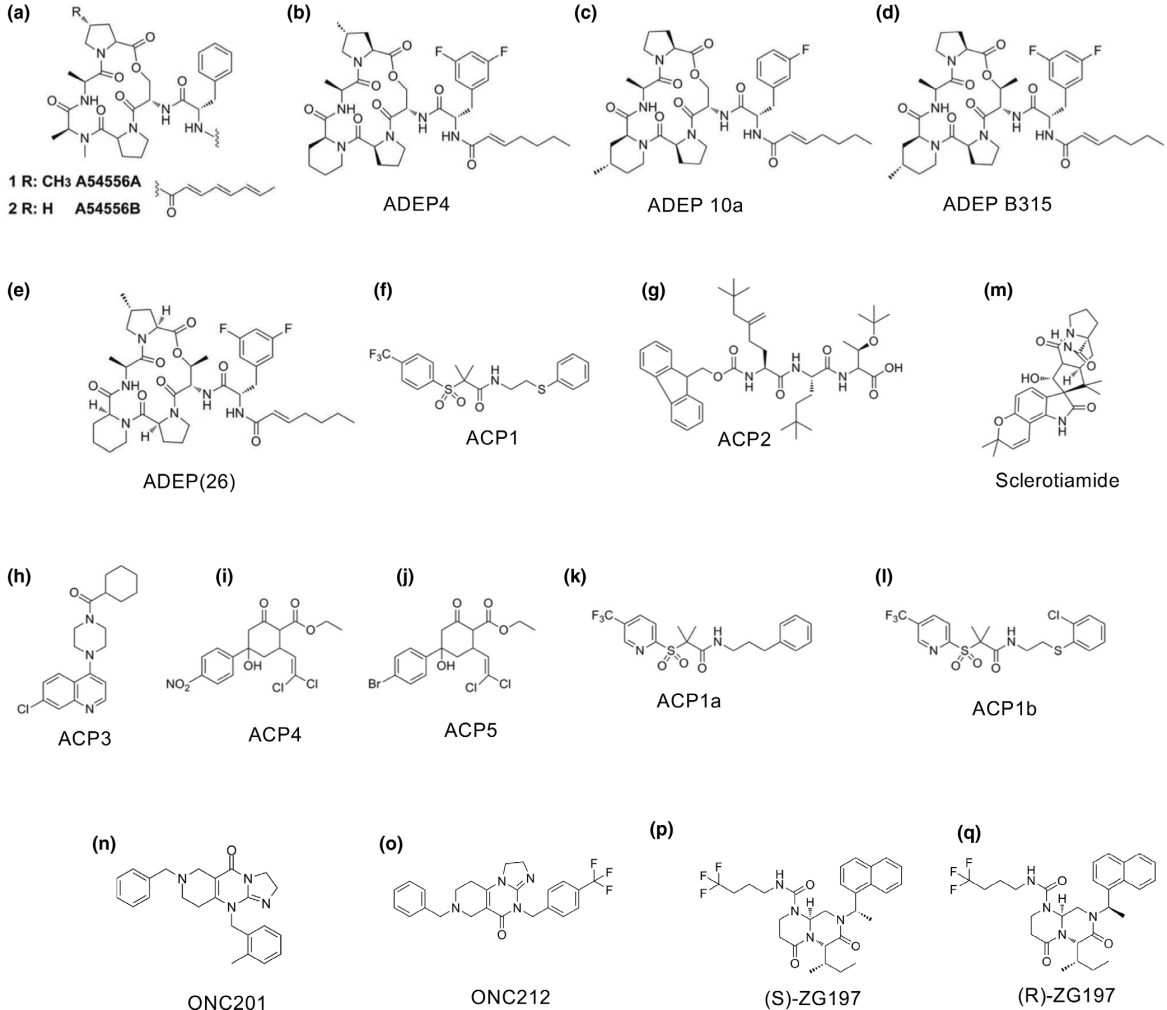
ClpP protease‐activator compounds. (a) A154556A and A54566B; (b) ADEP4; (c) ADEP10a; (d) ADEP B315; (e) ADEP(26); (f) ACP1; (g) ACP2; (h) ACP3; (i) ACP4; (j) ACP5; (k) ACP1a; (l) ACP1b; (m) Sclerotiamide; (n) ONC201; (o) ONC212; (p) (S)‐Z197; (q) (R)‐ZG197.

Protease activators are thought to be useful antibiotic compounds, as described in the previous section. Acyldepsipeptide (ADEP) has been isolated as an antibiotic from the culture fluid of *Streptomyces hawaiiensis* NRRL 15010, with a patent application: “A54556.” This patent reports that the drug exhibits in vitro activity against *Staphylococcus* and *Streptococcus*; however, no antibacterial effects were observed in in vivo mice. Furthermore, a depsipeptide antibiotic named “Enopeptin A,” which has antibacterial activity against *Staphylococcus aureus*, was identified from the culture fluid of *Streptomyces* bacteria (Osada et al., 1991). Subsequently, the structure of “Factor A” (hereinafter referred to as ADEP1), the main component of the A54556 complex, was determined (Figure 6a), and the derivatives were synthesized (Brötz‐Oesterhelt et al., 2005). Among these, ADEP4 (Figure 6b) exhibited the highest chemical stability and antibacterial activity (IC50 = ca. 0.05 μg/mL) against *S. aureus*. To identify the target of ADEP1, *E. coli*, which is defective in drug efflux pumps and sensitive to ADEP1 (forming filaments) under conditions that promote outer membrane permeation, was used for selection. The resistant transformants by plasmids in which the genes were introduced were isolated. The *clpP* gene or ClpP is required for ADEP‐dependent antibacterial activity. This study also demonstrated that ClpP binds directly to ADEP. Furthermore, ADEP4 analogs were synthesized, one of which was named 10a, and in vitro antibacterial activity (IC50 = ca. 0.04–0.16 μg/mL) against *Enterococci* was fourfold higher than that of the parent compound (Socha et al., 2010) (Figure 6c). Improved antibacterial activity was observed by replacing amino acids within the depsipeptide core structure of ADEP derivatives (Carney et al., 2014). ADEP B315 (Figure 6d), synthesized as a des‐methyl analog of ADEP4, was effective in the survival of mice infected with methicillin‐susceptible or methicillin‐resistant *S. aureus* strains (MIC = 0.024 μg/mL) (Arvanitis et al., 2016; Carney et al., 2014). ADEP(26) (Figure 6e), which has strong antibacterial activity and MIC with 0.0019–0.125 μg/mL for various bacteria, was also synthesized by modifying the acrocyclic core residue and *N*‐acyl side chain of the ADEP compound (Goodreid et al., 2016). This compound exhibits antibacterial activity against the gram‐negative bacteria *Neisseria meningitidis* and *Neisseria gonorrheae* and stronger antibacterial activity against the gram‐positive bacteria *S. aureus* and *Enterococcus faecalis* than ADEP‐like compounds synthesized to date. Additionally, the combination of ADEP4 and rifampicin efficiently killed persistent *S. aureus* in vitro and in a mouse model of chronic infection, raising the possibility that ADEP compounds could be used as antibacterial agents (Conlon et al., 2013).

As a result of large‐scale screening of compounds with similar activity to ADEP (Leung et al., 2011), using FITC‐casein as a substrate and increasing the protease activity against ClpP of *E. coli*, the five types of compounds named Activators of Self‐Compartmentalizing Proteases (ACP): ACP1, *N*‐1‐[2‐(phenylthio)ethyl]‐2‐methyl‐2‐{[5‐(trifluoromethyl)‐2‐pyridyl]sulfonyl}propanamide; ACP2, 3‐(tertbutoxy)‐2‐{[2‐[(5‐(tertbutoxy)‐2‐{[(9‐*H*‐9‐fluorenylmethoxy)carbonyl]amino}‐5‐oxopentanoyl)amino]‐3‐(tertbutylsulfanyl)propanoyl]amino}butanoic acid; ACP3, [4‐(7‐chloroquinolin‐4‐yl)piperazino](cyclohexyl)methane; ACP4, ethyl 2‐(2,2‐dichlorovinyl)‐4‐hydroxy‐4‐(3‐nitrophenyl)‐6‐oxocyclohexanecarboxylate; ACP5, ethyl 4‐(4‐bromophenyl)‐2‐(2,2‐dichlorovinyl)‐4‐hydroxy‐6‐oxocyclohexanecarboxylate were identified (Figure 6f–j). ACP1, ACP2, and ACP3 were obtained from MayBridge library, whereas ACP4 and ACP5 were obtained from ChemBridge library. When the activation ability of this compound was measured relative to the protease activity of ClpAP, the values were 0.53, 0.20, 0.10, 0.37, and 0.39, respectively, indicating that ACP1 had the highest activation ability. Subsequently, over 70 ACP analogs were synthesized, and highly active analogs ACP1a (Figure 6k) and ACP1b (Figure 6l) were obtained. When these compounds are administered to various bacterial cells, their effects do not necessarily correspond to their ability to activate proteases or their minimum lethal concentration (MBC), for example MBCs of ACP1, ACP2, ACP3, ACP4, ACP5, ACP1a and ACP1b are 64, 64, >256, 32, >256, 64 and 16 μg/mL, respectively, against *N. meningitidis*. In conclusion, there are differences in the ability of bacterial cells to take up compounds and their stability within bacterial cells or in the presence of an active site other than ClpP.

By screening a library of secondary metabolites from bacteria and fungi for compounds affecting ClpP activity, the non‐peptide compound sclerotiamide (Figure 6m) which EC_50_ for FITC‐casein was ca. 88 μM, a paraheruquamide‐related substance, was identified (Lavey et al., 2016). To improve the efficacy and coverage of ACP against gram‐negative bacteria, ACP derivatives have been developed and tested for susceptibility of clinical isolates of *N. meningitidis* and *N. gonorrheae*, resulting in significantly increased susceptibility (Binepal et al., 2020). ADEP28, an analog of ADEP4, and the novel low‐molecular‐weight compounds imipridone (ONC201 and ONC212) (Figure 6n,o) have been identified as substances that inhibit the proliferation of cancer cells by activating ClpP in human mitochondria (Graves et al., 2019; Ishizawa et al., 2019; Wong et al., 2018). The IC_50_ values of ONC201 and ONC212 for viability of OCI‐AML2 cells were reported as 2.4 μM and 76 nM, respectively (Ishizawa et al., 2019). ONC201 is also known to act as an antagonist of dopamine D2 receptors. (*R*)‐ and (*S*)‐ZG197 (Figure 6p,q) were obtained by structure‐based design of compounds with different activation abilities between human mitochondria and *S. aureus* ClpP (Wei et al., 2022). The EC_50_ values of (R)‐ZG197 or (S)‐ZG197 for *Sa*ClpP and *Hs*ClpP are1.5 ± 0.2 μM and 31.4 ± 0.6 μM, respectively or 1.4 ± 0.2 μM and > 100 μM, respectively. It is an effective antibiotic against *S. aureus* in both zebrafish and mouse skin infection models.

## 6 STRUCTURAL ANALYSIS OF CLP PROTEASE

**FIGURE 7:**
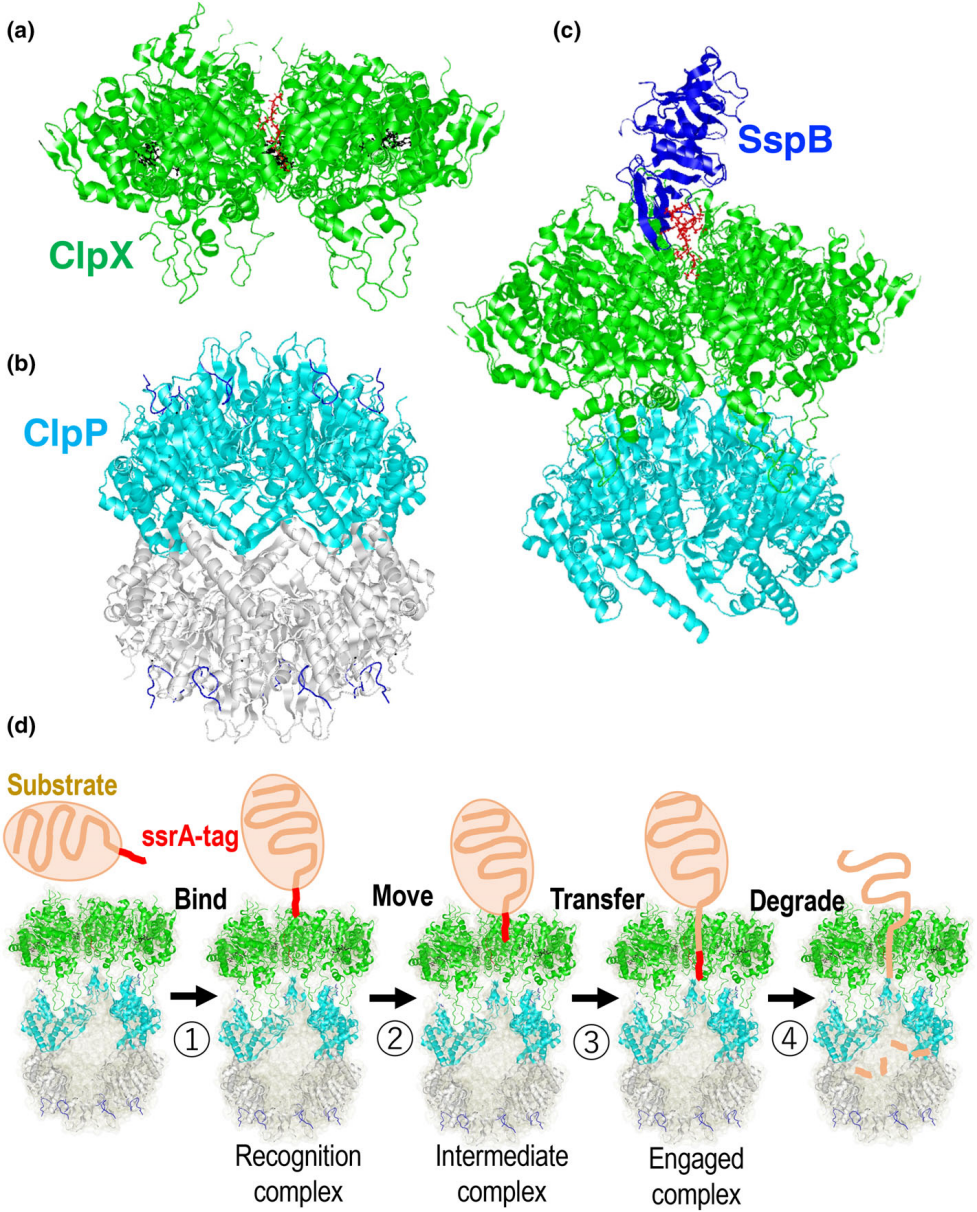
Clp protease structure. (a) The structure of ClpX (PDB: 6PP6) is shown by the ribbon model (yellow and green), and ATPγS and the ssrA tag of the substrate site are shown by the ball and stick model in black and red, respectively. (b) The structure of ClpP (PDB:6PPE) is shown by the ribbon model (light blue and white), and the ClpX IGF loop is by the ribbon model (blue). (c) The structure of ClpXP with SspB and (PDB:8ET3) (Ghanbarpour et al., 2023) is shown by the ribbon model for yellow and green (ClpX), or light blue (ClpP). The *ssrA* tag is shown by the ball and stick model (red), and SspB is shown by the ribbon model (blue). (d) Model of substrate binding, transfer, and unfolding by ClpXP. The ssrA tag (~20 residues) is recognized as a degron and binds to ClpX, and then an ATP degradation‐dependent power stroke moves the degron deep into the ClpX channel of the intermediate complex, where it is degraded while the substrate protein is unwound (Fei, Bell, Barkow, et al., 2020).

The crystal structure analysis of *E. coli* ClpP protease was conducted in 1997 (Wang et al., 1997). Subsequently, the crystal structures of ClpX from *Helicobacter pylori* and *E. coli* ClpA were reported (Guo et al., 2002; Kim & Kim, 2003). Since then, structural analyses of Clp adapters and proteases from various bacterial species have been performed, and detailed structural analyses have been included in other reviews (Mabanglo & Houry, 2022; Sauer et al., 2022). In this review, we provide an overview of the structural analysis of *E. coli* ClpXP. In 2020, a structural analysis using cryoelectron microscopy revealed the complex structure of ClpXP, including its substrate (Figure 7a,b) (Fei, Bell, Barkow, et al., 2020; Fei, Bell, Jenni, et al., 2020). The ClpX used here was ClpXΔN(62‐424), which lacks 61 residues at the N‐terminus, introduces mutations such as E185Q into the Walker motif to make it incapable of ATP degradation, and then adds six residues in tandem to ClpXΔN(62‐424). A His‐tag containing the TEV protease sequence was added to the C terminus for purification. This construct has been previously used to analyze the role of each subunit in the hexamer (Martin et al., 2005). In this construct, ClpP(1–193) has a propeptide attached to the N‐terminus, which is removed by autolysis, and a TEV‐His6 epitope, which is expressed in *E. coli* and purified (Kim et al., 2000). As a substrate, a GFP protein with an *ssrA* sequence (ENYALAA) added is used. After removing the His tags of these proteins with TEV protease, they were mixed with ATPγS to prepare frozen samples, which were observed using a cryoelectron microscope to obtain their structures. Protease degradation dynamics was determined by a fluorescence quenching assay using a 20‐residue *ssrA* degron fused to titin and modified with a black‐hole BHQ10 quencher (titin‐BQ‐ssrA) to detect the state of association between the substrate and ClpXP (Saunders et al., 2020). This suggests the existence of at least three substrate‐binding states: (i) initial recognition complex, (ii) intermediate complex, and (iii) engaged complex. The correspondence between these states and the cryoelectron microscopy structure was investigated, and a reaction model was proposed (Figure 7d). Furthermore, the structure of the *E. coli* ClpAPS complex, which is responsible for the degradation of proteins bearing an amino‐terminally destabilizing amino acid (N‐degron), was determined using cryoelectron microscopy, and the roles of the adapter proteins ClpA and ClpS were inferred (Kim et al., 2022). A recent report on the structure of ClpXP with an SspB adaptor showed that the initial step of ClpX binding pulls the substrate away from SspB and facilitates efficient degradation (Figure 7c) (Ghanbarpour et al., 2023).

## 7 ACTIVATION MECHANISM OF ClpP PROTEASE BY ANTIBIOTICS

**FIGURE 8:**
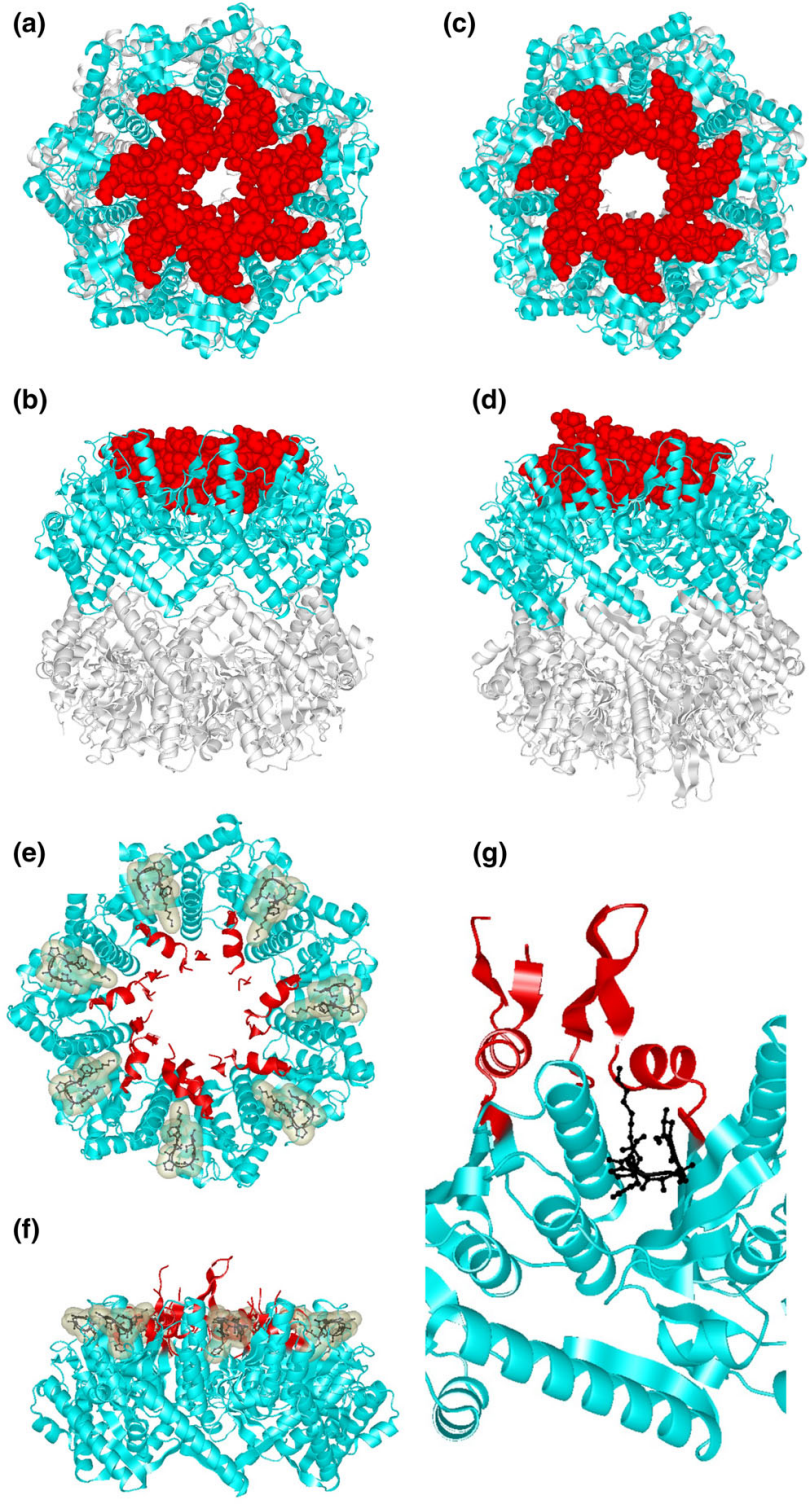
Structure of ClpP for protease activation. In the structure of ClpP (PDB:1TYF) (a, b) and the co‐crystal structure of ClpP with ADEP1 (PDB:3MT6) (c, d), 30 amino acids in the N‐terminal region of ClpP is shown by the space‐filling model (red) and the other residues are shown by ribbon model (light blue and white). (e, f) The structural data were the same as (c, d), but 30 amino acids of the N‐terminal region of ClpP are shown in red ClpP is shown by ribbon model and ADEP1 is shown by ball and stick model (black) with space filling. (g) A close‐up diagram of the binding site between ClpP and ADEP1 is shown.

Although ClpP is a serine protease, it does not exhibit protease activity unless it forms a tetradecamer and assembles with an unfoldase or a chaperone hexamer of ATPase. Small peptides can be degraded in the absence of unfoldases. In the presence of compounds such as ADEP derivatives, ClpP degrades proteins such as casein, even in the absence of unfoldase as mentioned in session 5 (Brötz‐Oesterhelt & Vorbach, 2021; Ye et al., 2016). As shown in Figure 7, ClpP forms a barrel‐shaped tetradecamer consisting of two overlapping heptamers. NTD (Figure 4b) binds to the substrate and is important for substrate recognition. The binding structure of ADEP1, which activates the ClpP protease, was solved in 2010 (Li et al., 2010). In the same year, the binding structures of *B. subtilis* ClpP, ADEP1, and ADEP2 were determined (Lee et al., 2010). In both structures, the pores at the substrate uptake sites widened (Figure 8). When this pore widened, the barrel‐shaped structure of the tetradecamer became slightly longer in the vertical direction (Figure 8b,d). ADEP1 bound to the interface of the heptameric ring structure (Figure 8e–g), stabilized the ring structure, and altered the structure of the N‐terminal hole. Based on the bound and unbound structures of ADEP1 in *E. coli* ClpP, allosteric communication during conformational changes was investigated by comparing the conjugation between residues in molecular dynamics simulations of the configuration (Dayananda et al., 2023). Community network analysis revealed a switch between open‐ and closed‐pore intraprotomer and interprotomer bonds caused by ADEP1 binding.

## 8 CONCLUSION

Clp proteases are important proteins that control bacterial metabolism by regulating the degradation of various gene products (Gottesman & Maurizi, 1992; Jenal & Hengge‐Aronis, 2003). This protease has been studied as a potential target for the development of novel antibiotics. Initially, the targets were substances that inhibit protease activity; however, substances that activate protease activity are now attracting attention. The natural antibiotic ADEP (Brötz‐Oesterhelt et al., 2005) was the first identified compound of this class. It is considered impractical for use as a medicine because of its complex structure, which makes it difficult to synthesize and exhibit antibacterial activity. However, through a rational design based on structural information, compounds with more specific activities can be synthesized, enabling their use as drugs. Furthermore, by activating the human mitochondrial Clp protease, it is possible to use the drug as an anticancer agent (Greer et al., 2022; Wedam et al., 2023).

## CONFLICT OF INTEREST STATEMENT

The authors declare that there are no conflicts of interest.
